# Metformin combined with aspirin significantly inhibit pancreatic cancer cell growth *in vitro* and *in vivo* by suppressing anti-apoptotic proteins Mcl-1 and Bcl-2

**DOI:** 10.18632/oncotarget.4126

**Published:** 2015-05-12

**Authors:** Wen Yue, Xi Zheng, Yong Lin, Chung S. Yang, Qing Xu, Darren Carpizo, Huarong Huang, Robert S. DiPaola, Xiang-Lin Tan

**Affiliations:** ^1^ Rutgers Cancer Institute of New Jersey, Rutgers, The State University of New Jersey, New Brunswick, NJ, USA; ^2^ Department of Chemical Biology, Ernest Mario School of Pharmacy, Rutgers, The State University of New Jersey, Piscataway, NJ, USA; ^3^ Department of Biostatistics, School of Public Health, Rutgers, The State University of New Jersey, Piscataway, NJ, USA; ^4^ Department of Oncology, Shanghai Tenth People's Hospital, Tongji University, School of Medicine, Shanghai, P. R. China; ^5^ Allan H. Conney Laboratory for Anticancer Research, Guangdong University of Technology, Guangzhou, P. R. China; ^6^ Department of Epidemiology, School of Public Health, Rutgers, The State University of New Jersey, Piscataway, NJ, USA

**Keywords:** metformin, aspirin, pancreatic cancer, Bcl-2 family member, apoptosis

## Abstract

Metformin and aspirin have been studied extensively as cancer preventive or therapeutic agents. However, the effects of their combination on pancreatic cancer cells have not been investigated. Herein, we evaluated the effects of metformin and aspirin, alone or in combination, on cell viability, migration, and apoptosis as well as the molecular changes in mTOR, STAT3 and apoptotic signaling pathways in PANC-1 and BxPC3 cells. Metformin and aspirin, at relatively low concentrations, demonstrated synergistically inhibitory effects on cell viability. Compared to the untreated control or individual drug, the combination of metformin and aspirin significantly inhibited cell migration and colony formation of both PANC-1 and BxPC-3 cells. Metformin combined with aspirin significantly inhibited the phosphorylation of mTOR and STAT3, and induced apoptosis as measured by caspase-3 and PARP cleavage. Remarkably, metformin combined with aspirin significantly downregulated the anti-apoptotic proteins Mcl-1 and Bcl-2, and upregulated the pro-apoptotic proteins Bim and Puma, as well as interrupted their interactions. The downregulation of Mcl-1 and Bcl-2 was independent of AMPK or STAT3 pathway but partially through mTOR signaling and proteasome degradation. In a PANC-1 xenograft mouse model, we demonstrated that the combination of metformin and aspirin significantly inhibited tumor growth and downregulated the protein expression of Mcl-1 and Bcl-2 in tumors. Taken together, the combination of metformin and aspirin significantly inhibited pancreatic cancer cell growth *in vitro* and *in vivo* by regulating the pro- and anti-apoptotic Bcl-2 family members, supporting the continued investigation of this two drug combination as chemopreventive or chemotherapeutic agents for pancreatic cancer.

## INTRODUCTION

Metformin and aspirin, two emerging candidate drugs of cancer chemoprevention, have been reported to decrease the risk of different types of cancers, including pancreatic cancer [1–6]. For example, a large case-control study found that diabetic patients who received metformin had a significant lower risk of pancreatic cancer (odds ratio = 0.38; 95% CI: 0.22–0.69; *P* = 0.001) comparing to those who did not, while insulin administration caused a higher risk of pancreatic cancer [1]. In a clinic-based case-control study involving 904 pancreatic cancer patients and 1224 controls, Tan *et al.* showed that aspirin use for 1 day per month or more frequently was associated with a significantly decreased risk of pancreatic cancer (odds ratio = 0.74, 95% CI: 0.60–0.91, *P* = 0.005) compared with never or less than 1 day per month [5]. In a pooled analysis of 25,570 patients in eight trials, Rothwell *et al.* recently reported that daily aspirin use reduced deaths due to several common cancers, including significant reductions in colorectal and pancreatic cancer deaths, with most benefit seen after 5 years of the scheduled trial treatment [7]. These investigations suggest that both metformin and aspirin have preventive effects against the development of pancreatic cancer.

In preclinical studies, metformin has been found to inhibit cell proliferation, migration and invasion in pancreatic cancer cells [8–10]. Metformin has also been shown to prevent the promotional effect of high-fat diet on N-nitrosobis(2-oxopropyl)amine (BOP)-induced pancreatic carcinogenesis in Syrian hamsters [11] and to inhibit the pancreatic cancer cell growth in xenograft models using athymic nude mice [10, 12, 13]. A recent study reported that metformin prevents the progression of pancreatic intraepithelial neoplasia (PanIN) to pancreatic ductal adenocarcinoma (PDAC) by targeting cancer stem cells and mTOR signaling in p48Cre/+.LSL-KrasG12D/+ transgenic mice [14]. Tan *et al.* also recently showed that metformin treatment may inhibit pancreatic tumorigenesis in the LSL-*Kras*^G12D/+^;*Trp53*^F2-10^ mice by modulating multiple molecular targets in signal transducer and activator of transcription 3 (STAT3) and nuclear factor kappa B (NFкB) inflammatory pathways [15]. Aspirin has been shown to suppress pancreatic cancer growth both *in vitro* and *in vivo* [16, 17]. Besides, a derivative of aspirin, nitric oxide–donating aspirin (NO-ASA), also showed chemopreventive effect in pancreatic cancer cell lines [18] and transgenic mice models [19].

Interestingly, metformin and aspirin have been found to share several underlying mechanisms on these protective effects. At the cellular level, metformin stimulates AMP-activated protein kinase (AMPK) activation by disrupting mitochondrial respiratory chain complex I and decreasing the ATP synthesis [20]. Recently, aspirin was also shown to inhibit the dephosphorylation of AMPK thus activating AMPK [21, 22]. AMPK maintains energy homeostasis by blocking protein synthesis and cell proliferation through inhibition of mTORC, which plays a pivotal role in cell survival and regulation of metabolism [23]. Metformin and aspirin can inhibit the mTOR signaling pathway through both AMPK-dependent and AMPK-independent mechanisms [21, 24, 25]. Given that persistent low-grade inflammation is an important factor for the development of pancreatic cancer, it is worth noting that two major inflammatory mediators, STAT3 and NFкB, also can be suppressed by metformin and aspirin [26–30]. These reported actions suggest possible better benefits in cancer prevention by using the combination of metformin and aspirin. However, this interesting possibility in pancreatic cancer has not been investigated.

Apoptotic cell death is tightly regulated by Bcl-2 family protein members. The anti-apoptotic Bcl-2 family proteins, such as Bcl-2 and Mcl-1, bind to their pro-apoptotic relatives and neutralize their pro-apoptotic activity [31]. Of the BH3-only proteins, Bim and Puma are the least selective, binding to all five anti-apoptotic proteins [32]. Cancer cells evolve diverse strategies to evade apoptosis by disturbing the intrinsic apoptotic pathway. They can achieve this goal by increasing the expression level of anti-apoptotic regulators such as Bcl-2 and Mcl-1, or downregulating pro-apoptotic proteins such as Bim and Puma [33]. Several Bcl-2 inhibitors have shown efficacy as chemotherapy agents in clinical trials [34]. However, there are some cancers that cannot be treated with these Bcl-2 inhibitors, in which the upregulation of Mcl-1 may play a key role [32]. Both metformin and aspirin can induce apoptosis in different cancer cells, including pancreatic cancer cells [9, 35–43]. However, the molecular mechanism for the apoptosis induced by metformin or aspirin has not yet been clearly elucidated.

In this study, we evaluated the effects of metformin and aspirin on cell viability, migration, and apoptosis in pancreatic cancer cell lines PANC-1 and BxPC3. We found that metformin combined with aspirin, at relatively low concentrations, demonstrated a synergistic effect on cell proliferation. Compared to the individual drug, we also found that the combination of metformin and aspirin had significantly stronger effects on the inhibition of colony formation and cell migration, as well as the modulation of the key molecular targets in AMPK, mTOR, STAT3 and NFкB pathways. Furthermore, the combination of metformin and aspirin led to apoptosis through downregulation of anti-apoptotic proteins Mcl-1 and Bcl-2 and upregulation of pro-apoptotic proteins Bim and Puma. We also showed that the downregulation of Mcl-1 and Bcl-2 was independent of AMPK or STAT3 pathway but partially through mTOR signaling and proteasome degradation. In a PANC-1 xenograft mouse model, we demonstrated that the combination of metformin and aspirin significantly inhibited tumor growth and downregulated the protein expression of Mcl-1 and Bcl-2 in tumors. These data provide rationale and experimental evidence for the combined use of metformin and aspirin in the prevention and/or treatment of pancreatic cancer.

## RESULTS

### Metformin and aspirin inhibited cell viability, colony formation and migration

**Figure 1:**
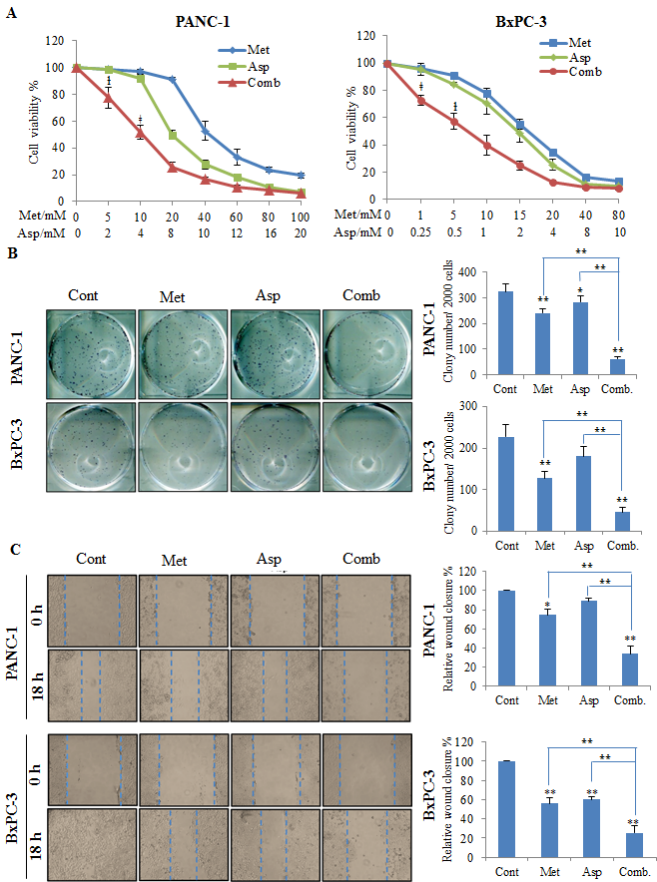
The combination of metformin and aspirin inhibit cell viability (A), colony formation (B) and migration (C) PANC-1 and BxPC-3 cells were treated with metformin and aspirin at indicated concentrations for 72 hours. Cell viability was evaluated by MTS assay. For colony formation assay, the treated and untreated cells were trypsinized and 1000 single viable cells were plated in 6-well plates and culture for additional 14 days. The colonies were stained with MTT and counted if they contain 50 cells or more. For wound-healing assay, cells were seeded in 12-well plates, and the wound was generated by scratching the surface of the plates with a pipette tip. Cells were then washed with PBS and incubated with metformin and aspirin at indicated concentration for 18 hours. Data are means ± SEM, *n* = 3 means of triplicate measures. ^ł^ *p* < 0.05, ^‡^ *p* < 0.01, the test for synergy; *p < 0.05, ^**^p < 0.01, compared to the untreated control or individual drug. Cont, control; Met, metformin; Asp, aspirin; Comb, the combination of metformin and aspirin.

To evaluate the potential synergy between metformin and aspirin on cell proliferation in pancreatic cancer cells, we first conducted MTS assay using different doses of metformin and aspirin in PANC-1 and BxPC-3 cells. We observed that the combination of metformin and aspirin caused a synergistic inhibition of cell viability in both PANC-1 and BxPC-3 cells, mainly in low dosages of both drugs [e.g., PANC-1 cells: 5 mM metformin and 2 mM aspirin, and 10 mM metformin and 4 mM aspirin (P for the test of synergy = 0.034 and 0.007, respectively); BxPC-3 cells: 1 mM metformin and 0.25 mM aspirin, and 5 mM metformin and 0.5 mM aspirin (P for the test of synergy = 0.004 and 0.045, respectively)] (Figure 1A). We then tested the effects of metformin and aspirin on colony formation of PANC-1 and BxPC-3 cells. The combination of metformin and aspirin significantly decreased the colonogenicity to about 20% compared to the control in both cell lines (Figure 1B). Furthermore, in anchorage-independent colony formation assay, metformin or aspirin alone could effectively inhibit the formation of colonies at dose of 5 mM or 2 mM, respectively, and this inhibition was even greater in cells treated with the combination of metformin and aspirin (Supplementary Figure S1). We also examined the effect of metformin and aspirin on cell migration by wound-healing assay, and observed that the combination metformin and aspirin acted more efficiently to inhibit the capacity of wound healing in both cell lines (Figure 1C).

### The effects of metformin and aspirin on different signaling pathways

**Figure 2:**
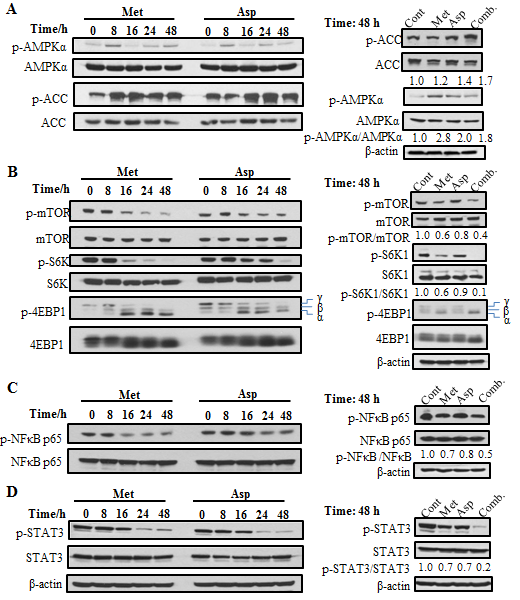
The effects of metformin and aspirin on different molecular signaling pathways including AMPK (A) mTOR (B), NFκB (C) and STAT3 (D) pathways PANC-1 cells were treated with 5 mM metformin or 2 mM aspirin for indicated time. Antibodies that detect the phosphorylated state of AMPKα at Tyr172, ACC at Ser79, mTOR at Ser2448, S6K at Thr389, 4E-BP1 at Thr37/46 NFκB p65 at Ser536 and STAT3 at Tyr705 were used. Values are expressed as fold of untreated control at that time and are means, *n* = 2 means of triplicate measures. Cont, control; Met, metformin; Asp, aspirin; Comb, the combination of metformin and aspirin.

To further investigate the mechanism responsible for the effects of the combination of metformin and aspirin, we evaluated the effects of metformin and aspirin on the expression and activation of selected signaling pathways. We first examined the effects of both drugs individually in PANC-1 cells on AMPK and acetyl-CoA carboxylase (ACC), which is a well-established AMPK downstream targeting enzyme [21]. The results showed that both metformin and aspirin could individually stimulate the phosphorylation of AMPK, especially on early time point (8 h) (Figure 2A, left panel). The activation of AMPK was accompanied by increased phosphorylation of ACC, which was continued to 48 h. When the cells were treated by the combination of metformin and aspirin, the phosphorylation of ACC was further erected (Figure 2A, right panel).

Since AMPK is a major regulator of mTOR pathway, we also examined the effects of metformin and aspirin on mTOR and its downstream targets, the ribosomal protein S6 kinase (S6K) and the eukaryotic initiation factor 4E-binding protein 1 (4E-BP1) in PANC-1 cells. Previous studies had demonstrated that both metformin and aspirin could inhibit the activity of mTORC1 [21, 24]. Consistent with those results, we also observed that treatment of metformin or aspirin led to a time-dependent decrease of the phosphorylation level of mTORC1 (Figure 2B, left panel). Furthermore, metformin or aspirin showed a strong inhibition of the phosphorylation of S6K. We also examined the phosphorylation of 4E-BP1 and detected three isoforms of 4E-BP1, which correspond to differentially phosphorylated forms of 4E-BP1 [44]. The slowest migrating band γ represents the hyper-phosphorylated form and the fastest migrating band α represents the hypo-phosphorylated form of the protein. Metformin or aspirin treatment caused a remarkable shift from the γ form to the α form, indicating a decrease of phosphorylation of 4E-BP1. Interestingly, the combination of metformin and aspirin showed stronger inhibition of the phosphorylation of mTOR, S6K and 4E-BP1, compared to the single agent (Figure 2B, right panel).

We then assessed the effects of metformin and aspirin on NFкB and STAT3 pathways in PANC-1 cells. Metformin and aspirin individually suppressed the phosphorylation of NFкB and STAT3 in a time-dependent manner (Figure 2C, 2D). Compared to a single agent, the combination of metformin and aspirin exerted a more pronounced effect on the suppression of NFкB (Figure 2C, right panel) and STAT3 (Figure 2D, right panel). These results showed that the combination of metformin and aspirin had significantly stronger effects on the above signaling pathways in PANC-1 cells, compared to the single agents.

### Metformin and aspirin induced apoptosis in pancreatic cancer cells

**Figure 3:**
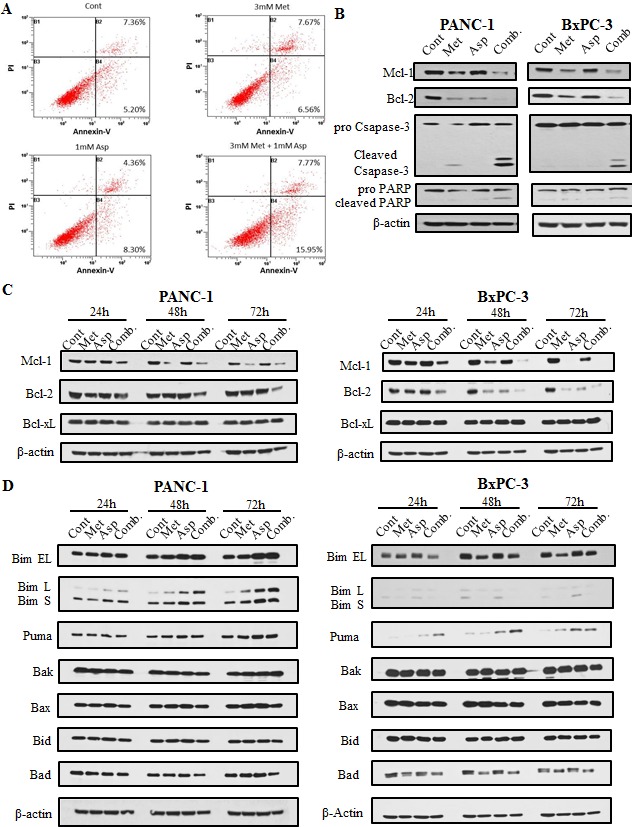
Metformin and aspirin induced apoptosis determined by analysis of apoptotic cells (A) and the expression of caspase-3 and PARP cleavage (B), as well as the expression of anti-apoptotic (C) and pro-apoptotic proteins (D) in pancreatic cancer cells A BxPC-3 cells were stained with annexin-V-FITC and PI following treatment with or without 3 mM metformin or 1 mM aspirin for 72 hours. Apoptosis was determined by flow cytometry, and the migrated distance was measured and statistically analyzed. (**B**, **C** and **D**) PANC-1 and BxPC-3 cells were treated with metformin and aspirin at the indicated concentration for 72 hours. Protein levels were detected by Western blotting using indicated antibodies, and β-actin was used as a loading control. Cont, control; Met, metformin; Asp, aspirin; Comb, the combination of metformin and aspirin.

To define the type of cell death induced by metformin and aspirin, we assessed the apoptotic cell death in BxPC-3 cells by flow cytometry using co-staining of Annexin-V and Propidium Iodide. Compared to the untreated control, treatment with metformin or aspirin induced a marginal to mediate increase of apoptotic cells (14.23% *vs*. 12.56%; 12.66% *vs*. 12.56%, respectively), and the combination of metformin and aspirin significantly increased the number of apoptotic cells (23.72%), as compared to the untreated control or individual drug (Figure 3A). We also determined the cleavage of caspase-3 and PARP, which are the hallmarks of apoptosis. Metformin alone induced a slight proteolytic cleavage of caspase-3 in PANC-1 cells, while the combination of metformin and aspirin significantly induced the cleavage of caspase-3 and PARP (Figure 3B, left panel). Similar results were also observed in BxPC-3 cells (Figure 3B, right panel).

### The effects of metformin and aspirin on anti-apoptotic and pro-apoptotic proteins

To further explore the mechanism of the apoptosis induced by metformin and aspirin, we investigated the protein levels of the Bcl-2 family members. We first examined the anti-apoptotic proteins Bcl-2, Mcl-1 and Bcl-xL in PANC-1 and BxPC-3 cells. The protein levels of Bcl-2 and Mcl-1 decreased in the cells treated with either metformin or aspirin, and these effects were significantly stronger in the cells treated with the combination of metformin and aspirin (Figure 3C). However, no significant change on the protein level of Bcl-xL was observed when the cells were treated with metformin and aspirin alone or a combination. We also determined the protein levels of pro-apoptotic Bcl-2 family members, including Bim, Puma, Bak, Bax, Bid and Bad, and most of them did not show significant changes (Figure 3D). However, the combination of metformin and aspirin significantly increased all the three isoforms of Bim in PANC-1 cells (Figure 3D, left panel) and Puma in BxPC3 cells (Figure 3D, right panel). These results suggested that the downregulation of Mcl-1 and Bcl-2, and the upregulation of Bim and Puma may be involved in the apoptosis induced by metformin and aspirin in pancreatic cancer cells.

### The effects of metformin and aspirin are independent of AMPK and STAT3

**Figure 4:**
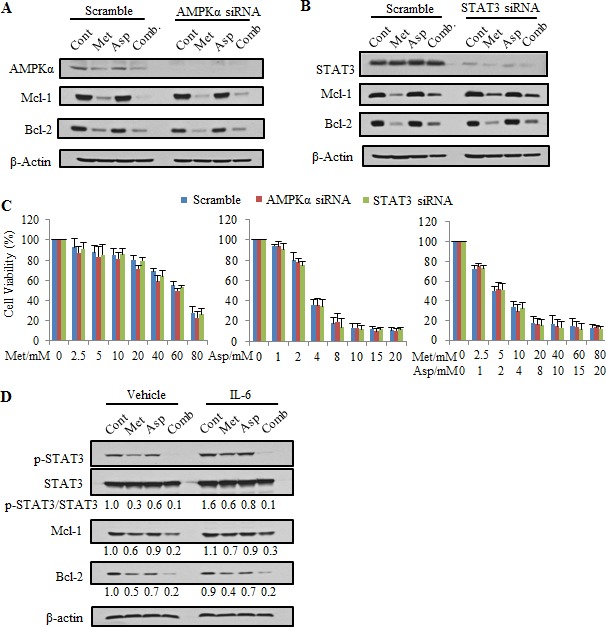
The synergistic effect of metformin and aspirin on cell viability and inhibition of Mcl-1 and Bcl-2 expression is independent of AMPK or STAT3 pathway (**A**, **B**) PANC-1 cells were transfected with scramble siRNA, AMPK targeted siRNA or Stat3 targeted siRNA, and then treated by 5 mM metformin and 2 mM aspirin for 72 hours. Whole cell lystaes were prepared and subjected to Western blot analysis. (**C**) PANC-1 cells were transfected with scramble siRNA, AMPK targeted siRNA or Stat3 targeted siRNA, and then treated by indicated concentrations of metformin and aspirin for 72 hours. Cell viability was evaluated by MTS assay. Data are means ± SEM, *n* = 3 means of triplicate measures. (**D**) BxPC-3 cells were treated by indicated agents (IL-6: 5 ng/mL, metformin: 5 mM, aspirin: 2 mM) for 48 hours. Whole cell lysates were prepared and subjected to Western blot analysis. Values are expressed as fold of untreated control and are means, *n* = 2 means of triplicate measures. Cont, control; Met, metformin; Asp, aspirin; Comb, the combination of metformin and aspirin.

To examine whether the downregulation of Mcl-1 and Bcl-2 is dependent on AMPK, we knocked down AMPKα to abrogate AMPK function (Figure 4A). After AMPKα was silenced, we still observed the downregulation of Mcl-1 and Bcl-2 induced by metformin and aspirin, indicating that the inhibitory effect of metformin and aspirin on Mcl-1 and Bcl-2 is independent of the activity of AMPK. We observed that both metformin and aspirin significantly inhibited the activity of STAT3, and STAT3 was previously showed to regulate Mcl-1 and Bcl-2 in various cancer cell lines [35, 45, 46]. Therefore, we determined whether metformin and aspirin regulated Mcl-1 and Bcl-2 through STAT3 inhibition. In our experiment, knockdown of STAT3 did not alter the protein levels of Mcl-1 and Bcl-2, and metformin and aspirin could still decrease Mcl-1 and Bcl-2 protein levels in the siRNA transfected STAT3-silenced PANC-1 cells (Figure 4B). Furthermore, we assessed the cell viability in AMPKα or STAT3 knockdown cells upon metformin and aspirin treatment (Figure 4C). Compared to the control, knockdown of AMPKα or STAT3 has no significant effect on the inhibition of cell viability by metformin and aspirin. These results suggested that the inhibitory effects of metformin and aspirin on cell proliferation and the protein expression of Mcl-1 and Bcl-2 are independent of AMPK and STAT3 in PANC-1 cells.

To further evaluate the role of STAT3 in the regulation of Mcl-1 and Bcl-2, we examined whether IL-6, a STAT3 activator, could revert the downregulation of Mcl-1 and Bcl-2 in the BxPC3 cells treated with metformin and/or aspirin. We observed that IL-6 alone significantly induced the phosphorylation of STAT3, but could not induce the protein expression of Mcl-1 and Bcl-2 (Figure 4D). In addition, the inhibitory effects of metformin and aspirin on the protein expression of Mcl-1 and Bcl-2 in BxPC3 cells could not be compensated when the cells were simultaneously treated with IL-6 (Figure 4D). These results further suggested that the inhibitory effect of metformin and aspirin on Mcl-1 and Bcl-2 is independent of STAT3 inhibition.

### Metformin and aspirin downregulated Mcl-1 and Bcl-2 through proteasome degradation and mTOR pathway

**Figure 5:**
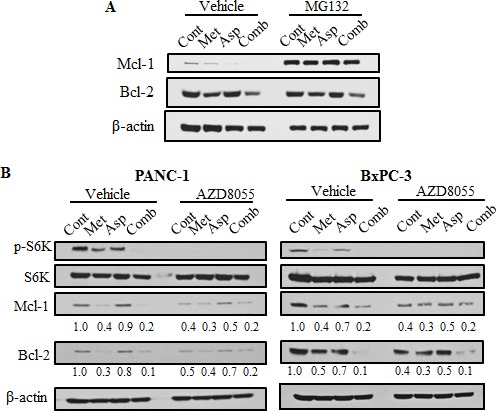
Metformin and aspirin decrease Mcl-1 and Bcl-2 expression through both mTOR pathway and proteasome degradation (**A**) PANC-1 cells were treated by indicated agents (MG132: 5 μM, metformin: 5 mM, aspirin: 2 mM) for 48 hours. Whole cell lysates were prepared and subjected to Western blot analysis. β-actin was used as a loading control. (**B**) PANC-1 cells were treated by indicated agents (AZD8055: 1 μM, metformin: 5 mM, aspirin: 2 mM) for 48 hours. Whole cell lysates were prepared and subjected to Western blot analysis. β-actin was used as a loading control. Values are expressed as fold of untreated control and are means, *n* = 2 means of triplicate measures. Cont, control; Met, metformin; Asp, aspirin; Comb, the combination of metformin and aspirin.

Because Mcl-1 is mainly regulated by the ubiquitin-dependent proteasome degradation [47], we examined whether the proteasome inhibitor MG132 could reverse the decreasing of Mcl-1and Bcl-2 upon the treatment of metformin and aspirin. MG132 could completely reverse the metformin- or aspirin-mediated downregulation of Mcl-1, but not Bcl-2 (Figure 5A). The results suggest that the downregulation of Mcl-1 by metformin and aspirin is through proteasome degradation, and the different mechanisms might be involved in the regulation of Mcl-1 and Bcl-2 by metformin and aspirin.

Mcl-1 has also been reported to be transcriptional regulated by mTORC1 [48], therefore, we determined whether the regulation of Mcl-1 and Bcl-2 by metformin and aspirin is dependent on the mTORC1 activity. We observed that AZD8055, a mTORC1 inhibitor, induced a comparable reduction of the phosphorylation of S6K, resulting in inhibition of Mcl-1 and Bcl-2 expression (Figure 5B). However, AZD8055 could not further enhance the downregulation of Mcl-1 and Bcl-2 induced by metformin and aspirin (Figure 5B). These results indicated that metformin and aspirin may regulate the expression of Mcl-1 and Bcl-2 partially through mTOR pathway.

### The combination of metformin and aspirin disrupted the balance between Mcl-1 and Bim/Puma

**Figure 6:**
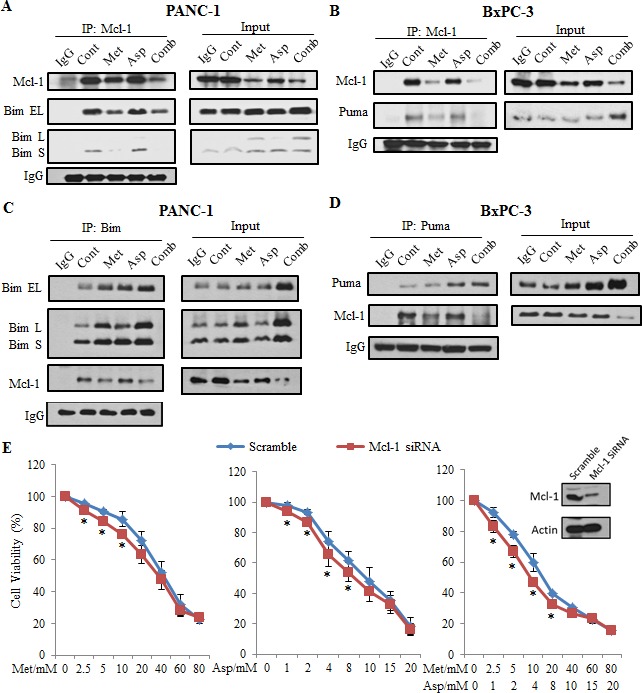
Metformin and aspirin disrupt Mcl-1: Bim and Mcl-1: Puma complexes and knock-down of Mcl-1 sensitized cells to metformin and aspirin (**A**) PANC-1 cells were treated by indicated agents (metformin: 5 mM, aspirin: 2 mM) for 48 hours. Mcl-1 was immunoprecipitated from the whole cell lysates and analyzed for the presence of Mcl-1 and Bim. (**B**) BxPc-3 cells were treated by indicated agents (metformin: 3 mM, aspirin: 1 mM) for 48 hours. Mcl-1 was immunoprecipitated from the whole cell lysates and analyzed for the presence of Mcl-1 and Puma. (**C**) PANC-1 cells were treated by indicated agents (metformin: 5 mM, aspirin: 2 mM) for 48 hours. Bim was immunoprecipitated from the whole cell lysates and analyzed for the presence of Mcl-1 and Bim. (**D**) BxPc-3 cells were treated by indicated agents (metformin: 3 mM, aspirin: 1 mM) for 48 hours. Puma was immunoprecipitated from the whole cell lysates and analyzed for the presence of Mcl-1 and Puma. (**E**) PANC-1 cells were transfected with scramble siRNA or Mcl-1 targeted siRNA. Twenty-four hours later, cells were treated by indicated concentrations of metformin and aspirin for additional 72 hours. Cell viability was evaluated by MTS assay. Data are means ± SEM, *n* = 3 means of triplicate measures. *p < 0.05, compared to the cells transfected with scramble siRNA. Cont, control; Met, metformin; Asp, aspirin; Comb, the combination of metformin and aspirin.

To further elucidate the mechanism by which the combination of metformin and aspirin induced apoptosis, we performed immunoprecipitation to examine the interaction of Mcl-1 with Bim in PANC-1 cells and Puma in BXPC-3 cells. We first immunoprecipitated endogenous Mcl-1 protein from these cells and determined its binding to Bim using anti-Bim antibody. We observed that Bim-EL or Bim-S that binding to Mcl-1 significantly decreased after the treatment of metformin and aspirin (Figure 6A). Similar effects of metformin and aspirin on the interaction between Mcl-1 and Puma were also observed in BxPC3 cells (Figure 6B). Since the Mcl-1 protein expression significantly decreased in the cells treated with metformin and aspirin, we could not exclude the possibility that the decreases of Bim and Puma levels were due to the down-regulation of Mcl-1. To further clarify the treatment effects on the interaction between Mcl-1 and Bim/Puma, we performed the reverse immunoprecipitation by immunoprecipitating endogenous Bim or Puma and immunobloting with anti-Mcl-1 antibody. Although the immunoprecipitated Bim or Puma in PANC-1 or BxPC3 cells increased, the antiapoptotic proteins Mcl-1 interacting with them significantly decreased, indicating an increase of free Bim or Puma which will lead to apoptosis (Figure 6C, 6D). In addition, we observed that metformin and aspirin could also decrease the interaction between Bcl-2 and Puma in BxPC3 cells (Supplementary Figure S2), but we were not able to detect the interaction between Bcl-2 and Bim in PANC-1 cells (data not shown). Furthermore, knockdown of Mcl-1 could sensitize the PANC-1 cells to metformin and aspirin (Figure 6E), indicating that the downregulation of Mcl-1 played a pivotal role in the apoptosis induced by metformin and aspirin alone or in combination.

### The combination of metformin and aspirin inhibited the growth of PANC-1 xenograft tumors in immunodeficient mice

**Figure 7:**
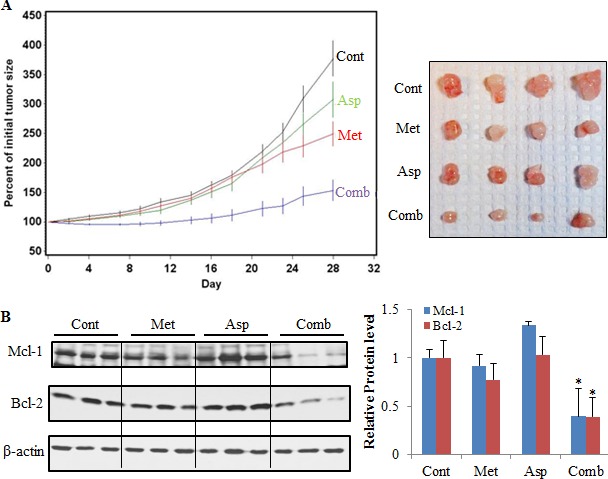
The combination of metformin and aspirin inhibited tumor growth and downregulated the protein expression of Mcl-1 and Bcl-2 in a PANC-1 xenograft mouse model (**A**) The combination of metformin and aspirin significantly inhibited pancreatic tumor growth. 2 × 10^6^ PANC-1 cells suspended in 50% Matrigel in DMEM medium were injected subcutaneously into the right flank of the SCID mice. The mice with a tumor (0.5–1.0 cm wide and 0.5–1.0 cm long) were randomized and injected by i.p. with vehicle (5 *μ*L/g body weight), metformin (200 mg/kg), aspirin (60 mg/kg), or metformin (200 mg/kg) plus aspirin (60 mg/kg) three times a week for 28 days (*n* = 10 per group). The tumor size (length and width) was measured before each i.p. injection and tumor volume was calculated by using the formula length × width × width/2. The left panel shows changes of the percent of initial tumor volume in each group by Day, and the right panel shows representative images of the tumors by group. Values are means ± SE, *n* = 10. (**B**) The combination of metformin and aspirin downregulated the protein expression of Mcl-1 and Bcl-2 in tumors. The protein expression of Mcl-1 and Bcl-2 were analyzed in tumors by Western blot with indicated antibodies. The left panel shows representative results of Western blot, and the right panel shows densitometry analyses of the relative protein expression. Values are expressed as fold of the vehicle-treated control and are means ± SE, *n* = 10. *p < 0.05, compared to vehicle-treated control. Cont, control; Met, metformin; Asp, aspirin; Comb, the combination of metformin and aspirin.

The combination of metformin and aspirin had a strong inhibitory effect on tumor growth, whereas injection of aspirin alone had only a small inhibitory effect on tumor growth, and administration of metformin alone had a moderate inhibitory effect on tumor growth (Figure 7A). The quadratic trends in tumor growth for all the treatment groups were significantly different from the vehicle control group (Asp *vs*. Cont: *P* = 0.009, Met *vs*. Cont: *P* < 0.0001, Comb *vs*. Cont: *P* < 0.0001). The linear trend for the metformin alone and aspirin alone were not significantly different from the control group (*P* = 0.815 and 0.272, respectively), while the linear trend in tumor growth for the combination of metformin and aspirin was significantly different from that for any other groups (*P* ≤ 0.02). At the last time point (Day 28) when the mice was sacrificed, the mean ± standard error (SE) for the percent of initial tumor volume was 377.2 ± 29.8% for the vehicle treated control group, 307.3 ± 30.2% for the aspirin alone, 249.2 ± 20.5% for the metformin alone, 153.4 ± 17.6% for the combination of metformin and aspirin. The percent of initial tumor volume at the last time point for the combination group was statistically significantly lower than that for any other groups after the Tukey's multiplicity adjustments (Comb *vs*. Cont: *P* < 0.0001, Comb *vs*. Asp: *P* = 0.0006, Comb *vs*. Met: *P* = 0.049) (Figure 7A). No statistically significant difference in the percent of initial body weight between the control group and any of the treatment groups was observed during the experiments (Supplementary Figure S3).

Furthermore, we determined the protein expression of Mcl-1 and Bcl-2 in the mice tumors, and demonstrated that the combination of metformin and aspirin significantly decreased the protein levels of both Mcl-1 and Bcl-2 *in vivo* (Figure 7B). However, we did not detect significant changes on Mcl-1 and Bcl-2 protein expression in the mice treated with metformin or aspirin alone (Figure 7B).

## DISCUSSION

In this study, we observed that metformin and aspirin exhibited synergistic effect on the cell proliferation inhibition. Compared to the individual agents, we showed that the combination of metformin and aspirin had significantly stronger effects on the inhibition of colony formation and cell migration in PANC-1 and BxPC-3 cells. These effects of metformin and aspirin are independent of AMPK, which is a well-established upstream target of metformin. Our results also showed that metformin combined with aspirin could significantly downregulate anti-apoptotic proteins Mcl-1 and Bcl-2 and upregulate pro-apoptotic proteins Bim and Puma. The immunoprecipitation experiments revealed that metformin alone or combined with aspirin abrogated the interaction between Mcl-1 and Bim corresponding to the increase of Bim in PANC-1 cells and disrupted the interaction between Mcl-1 and Puma corresponding to the increase of Puma in BxPC3 cells. In a PANC-1 xenograft mouse model, we further demonstrated that the combination of metformin and aspirin significantly inhibited tumor growth and downregulated the protein expression of Mcl-1 and Bcl-2 in tumors. These results suggested that the combination of metformin and aspirin could significantly inhibit pancreatic cancer cell growth *in vitro* and *in vivo* though suppressing anti-apoptotic protein Mcl-1 and Bcl-2, and increasing pro-apoptotic proteins Bim and Puma, as well as disrupting their interactions in pancreatic cancer cells.

To the best of our knowledge, this is the first study to show that metformin combined with aspirin suppress the expression of anti-apoptotic Bcl-2 family member Mcl-1 in pancreatic cancer cells. Mcl-1 amplification is one of the most common genetic aberrations observed in human cancers, including pancreatic cancer [34, 47], indicating Mcl-1 plays a key role in pancreatic tumorigenesis. Studies also showed that Mcl-1 highly expressed in pancreatic cancer cells which was related to the resistance to chemotherapeutic drugs [49]. *In vitro*, we observed that the protein level of Mcl-1 was reduced by either metformin or aspirin and the combination of two drugs further decreased the Mcl-1 protein level. Since Mcl-1 is strongly regulated by proteasome degradation, we compared the effects of metformin and aspirin in the absence and presence of the proteasome inhibitor MG132. Our results showed that metformin and aspirin failed to decrease Mcl-1 protein level in the presence of MG132, indicating that metformin and aspirin induced proteasome degradation of Mcl-1. In contrast, MG132 failed to rescue the reduction of Bcl-2 induced by metformin and aspirin. These findings imply that metformin and aspirin regulate the expression level of Mcl-1 (but not Bcl-2) partially through proteasome degradation.

The mTOR signaling pathway has been reported to regulate Mcl-1 at both transcriptional and translational levels [48, 50]. mTOR also can indirectly regulate proteasome degradation of Mcl-1 by repressing GSK3 [51]. In our study, AZD8055, an mTOR inhibitor, significantly decreased the phosphorylation of S6K, resulting in repression of the expression of Mcl-1 in the two pancreatic cancer cell lines. However, AZD8055 could not further enhance the downregulation of Mcl-1 induced by metformin and aspirin. These results suggest that the regulation of Mcl-1by metformin and aspirin are at least partially through mTOR pathway. Given that metformin and aspirin could downregulate Mcl-1 through protein degradation (Figure 5A), it is also possible that mTOR might partially contribute to the proteasome-dependent degradation of Mcl-1 induced by metformin and aspirin. To the best of our knowledge, we found, for the first time, that the expression of Bcl-2 was downregulated upon AZD8055 treatment, raising the possibility that Bcl-2 is also regulated by mTOR. More studies are warranted to determine whether and how mTOR regulates Bcl-2 at transcription or translational level.

AMPK has been shown to be a main mediator of metformin in various cancer cell lines. In our study, although AMPK could be activated by both metformin and aspirin, which is consistent with other previous reports, AMPK activation is not indispensable for the anti-proliferation effect of metformin and aspirin. When AMPKα was silenced by specific siRNA, the survival curves of the cells treated by metformin, aspirin or the combination did not show significant difference. Furthermore, the downregulation of Mcl-1 and Bcl-2 by metformin and aspirin were still observed in the absence of AMPKα, indicating that the regulation of Mcl-1 and Bcl-2 by metformin and aspirin is independent of AMPK activation.

STAT3 is a key player in inflammation-related tumorigenesis, including pancreatic cancer, by promoting tumor cell proliferation and survival [46]. We observed that metformin and aspirin significantly inhibited the phosphorylation of STAT3 in PANC-1 and BxPC-3 cells. This is consistent with the previous findings that both metformin and aspirin inhibited the transcriptional activation of STAT3 in various cancer cell lines [26–28, 35, 52–54]. However, in our study, knockdown of STAT3 did not change the sensitivity of the cells to metformin and aspirin. Moreover, the protein levels of Mcl-1 or Bcl-2 did not change in STAT3-silenced cells. These results suggest that the inhibition of STAT3 is not involved in the proliferation inhibitory effect or the downregulation of Mcl-1 and Bcl-2 induced by metformin and aspirin in the studied pancreatic cancer cells.

We found that metformin and aspirin-induced apoptosis is mediated by the downregulation of antiapoptotic Bcl-2 family member Mcl-1 and Bcl-2, the upregulation of the proapoptotic BH3-only proteins Bim and Puma as well as the interruption of their interactions. Mcl-1 contributes to oncogenesis by both promoting apoptotic resistance and supporting high-rate proliferation of cancer cells, which makes it a critical molecule in cancer initiation and progression [32, 47]. Consistent with this, inhibition of Mcl-1 by siRNA sensitized PANC-1 cells to metformin and aspirin, further emphasizing the important role of Mcl-1 in metformin and aspirin-induced apoptosis. Among BH3-only proteins, Bim and Puma would be more efficient because of their ability to potently bind all the antiapoptotic proteins of Bcl-2 family, including Mcl-1 [33]. After the PANC-1 cells were treated with metformin and aspirin, the protein levels of Bim were increased, and the interaction between Mcl-1 and Bim/Puma was decreased. In BxPC-3 cells, we also observed that metformin and aspirin increased the protein level of Puma and abrogated the interaction between Puma and Mcl-1. These results are consistent with the idea that that particular BH3-only proteins drive the responses to specific cytotoxic insults, with Bim and Puma often being essential [31]. Increased levels of Puma and Bim may initiate apoptosis through the activation of Bax and Bak [55]. We did not find the upregulation of Bax and Bak after the treatment with metformin and aspirin, but more evidence are needed to determine whether Bax and Bak are activated in pancreatic cancer cells treated with metformin and aspirin.

In this study, we evaluated, for the first time, the effects of the combination of metformin and aspirin at relatively low concentrations in pancreatic cancer cell lines. The published *in vitro* studies on anti-cancer effects of metformin had used the dosages of metformin ranged from 1 mM to 50 mM. Although the doses of metformin we used is still higher than the physiologically achievable plasma concentration, metformin has been reported to accumulate within tissues at concentrations several folds higher than in blood [56]. In addition, metformin was also reported to accumulate in the mitochondrial matrix with a concentration higher than 20 mM due to the positive mitochondrial membrane potential [57, 58]. The plasma salicylate (the *in vivo* metabolite of aspirin) concentrations in human are 1~2 mM, which is similar with the concentrations we used *in vitro*.

Furthermore, we utilized a PANC-1 xenograft model to determine the effects of the combination of metformin and aspirin at physiologically achievable concentrations (the human equivalent doses of 416.9 mg/day and 125 mg/day for metformin and aspirin, respectively) on tumor growth. The results showed that the combination had significantly stronger effects on the inhibition of the pancreatic tumor growth than the individual agent. Additionally, the combination of metformin and aspirin significantly suppressed the protein expression of Mcl-1 and Bcl-2 in tumors, while no significant changes on Mcl-1 and Bcl-2 protein expression were observed in the mice treated with metformin or aspirin alone. These data suggested that the combination of metformin and aspirin within therapeutic dose range had better benefits on inhibition of pancreatic cancer cell growth, compared to metformin or aspirin alone, indicating a novel approach for pancreatic cancer prevention using the combination of metformin and aspirin.

In conclusion, the combination of metformin and aspirin exert synergistic cytotoxicity in pancreatic cancer cell lines and significantly inhibitory effect on *in vivo* tumor growth by inducing apoptotic cell death through the downregulation of anti-apoptotic Bcl-2 family member Mcl-1 and Bcl-2, and the upregulation/activation of the pro-apoptotic BH3-only proteins Bim and Puma. It is of interest to explore the possibility of using the combination of the two drugs to prevent the development of pancreatic cancer. The ongoing clinical trials on metformin and pancreatic cancer are focusing on assessing the efficacy of metformin in combination with standard and experimental therapeutics in advanced pancreatic cancer patients. Our study provides a rationale for the potential clinical trials that combine metformin with aspirin to assess the preventive effect on pancreatic cancer in high-risk population.

## MATERIALS AND METHODS

### Cell lines

Human pancreatic cancer cell lines PANC-1 and BxPC-3 were purchased from the American Type Culture Collection (Manassas, VA, USA). PANC-1 cells were maintained in Dulbecco's Modified Eagle's Media (DMEM) (Sigma-Aldrich, St Louis, MO, USA) supplemented with 10% heat-inactivated fetal bovine serum (FBS) (Sigma-Aldrich, St Louis, MO, USA). BxPC-3 cells were maintained in RPMI-1640 medium (Sigma-Aldrich, St Louis, MO, USA) containing 10% FBS. Both cell lines were grown in an incubator in 5% CO_2_ at 37°C.

### Antibodies and reagents

Antibodies against phosphor-AMPKα, AMPKα, phosphor-ACC, ACC, phosphor-mTOR, mTOR, phosphor-p70S6K, p70S6K, phosphor-4E-BP1, 4E-BP1, phorphor-NFκB p65, NFκB p65, IκBα, COX2, phosphor-STAT3, STAT3, Mcl-1, Bcl-xL, Bim, Bak, Bax, Bid, Puma and Bad were purchased from Cell Signaling Technology (Beverly, MA, USA). Antibodies against Bcl-2 and Protein A/G Agarose beads were purchased from Santa Cruz Biotechnology (Santa Cruz, CA, USA). Metformin and aspirin as well as interleukin-6 (IL-6) were purchased from Sigma-Aldrich (St Louis, MO, USA). AZD-8055 was purchase from Invitrogen (Carlsbad, CA, USA).

### Cell viability

Cells (5000 cells per well) were plated into 96-well plates for 24 hours and then treated with indicated concentrations of metformin and/or aspirin for additional 72 hours. Cell viability was assayed using CellTiter96 AQ nonradioactive Cell proliferation kit (Promega, Fitchburg, WI, USA) according to the manufacturer's instructions. The percentages of surviving cells from each group were calculated relative to controls. Controls were defined as 100% survival.

### Colony formation

PANC-1 and BxPC-3 cells (5 × 10^5^ cells per well) were seeded in 6-well plates and treated with indicated agents (5 mM metformin, 2 mM aspirin for PANC-1 cells; 1 mM metformin, 0.25 mM aspirin for BxPC-3 cells) for 3 days. Cells were trypsinized and 1000 single viable cells were plated in a well of 6-well plate. All the cells were cultured for additional 14 days and stained with 0.5 mg/mL MTT. The numbers of colonies containing 50 cells or more were counted.

### Wound-healing motility assay

Cells (1 × 10^5^ cells per well) were seeded into 12-well plate. The wound was generated when the cells reached 90%-95% confluency by scratching the surface of the plates with a pipette tip. Cells were washed thrice with PBS to remove cell debris and incubated with indicated agents (5 mM metformin, 2 mM aspirin for PANC-1 cells; 3 mM metformin, 1 mM aspirin for BxPC-3 cells) for 18 hours and then photographed with phase-contrast microscope. The migrated distance for each group was measured and statistically analyzed.

### Apoptosis assay

Apoptosis was determined by using an Annexin V-FITC/PI apoptosis detection kit (BD Biosciences) according to the manufacturers’ instructions. Briefly, the untreated and treated cells were washed with PBS buffer and gently suspended in Annexin V binding buffer and incubated with Annexin V-FITC (20 μg/mL) and PI (20 μg/mL) for 15 minutes in the dark. Flow cytometry analysis was performed using Cellquest software.

### Western blotting

The treated and untreated cells were rinsed twice with ice-cold PBS and extracted on ice with cell lysis buffer (Cell signaling, Beverly, MA, USA) which contains 20 mM Tris-Hcl (pH 7.5), 1% Triton, 150 mM NaCl, 1 mM EDTA, 1 mM EGTA, 1 mM Na3VO4, 2.5 mM Na pyrophosphate, 1 mM beta-glycerophosphate, 1 μg/mL leupeptin and 1 mM PMSF. The protein concentrations of lysates are determined with BCA Protein Assay Kit (Thermo scientific, Waltham, MA, USA). A stock of the extract was made in 1 × Laemmli buffer (Bio-Rad, Hercules, CA, USA) and stored at −20°C for western blot analysis. 20 μg of total proteins from each sample were loaded and separated on a gradient 4-20% polyacrylamide gel and transferred to polyvinylidene difluoride (PVDF) membrane. Membranes were blocked with 5% fat-free milk in Tris-buffered saline-Tween 20 (TBST, 20mM Tris, pH 7.6, 137 mM NaCl, and 0.1% Tween 20) for 1 hour at room temperature, followed by an overnight incubation at 4°C with the primary antibodies. Blots were subsequently washed three times with TBST and then incubated with the appropriate HRP-conjugated secondary antibodies for 1 hour at room temperature. After three additional TBST washes, the immunoreactive bands were visualized by enhanced chemiluminescence (Thermo Fisher, Rockford, IL USA) according to the manufacturer's instructions. The levels of β-actin were estimated to check for equal samples loading. Films were scanned and band densities were quantified with densitometric analysis using ImageJ software (NIH).

### Gene knockdown using siRNA

The siRNAs to AMPKα, STAT3 and Mcl-1 as well as the control scramble siRNA were purchased from Santa Cruz Biotechnology (Santa Cruz, CA, USA). The siRNA transfection was carried out using Lipofectamine 2000 reagent (Invitrogen, Carlsbad, CA, USA) according to the manufacturer's instructions. After 48 hours, the transfected cells were treated with metformin and aspirin, alone or in combination for additional 72 hours. Cell viability assay and Western blotting were performed as described above to determine the effects of metformin and aspirin on cell proliferation and specific molecular changes, respectively.

### Protein immunoprecipitation

After indicated treatments, cells were collected and lysed in cell lysis buffer with protease inhibitor cocktail on ice. 20 μL of protein A/G sepharose beads and 1 μg indicated antibody were added to the cell lysates containing 500 μg total proteins. Following overnight incubation with gentle rocking at 4°C, the beads were washed with PBS buffer for 4 times and analyzed by Western blotting.

### *In vivo* experiments

Female severe combined immunodeficient (SCID) mice (6–7 weeks old) were obtained from Taconic Farms Inc (Germantown, NY, USA). The animals were housed in sterile filter-capped microisolator cages and provided with sterilized food and water. PANC-1 cells (2×10^6^ cells/0.1 mL/mouse) suspended in 50% Matrigel (Collaborative Research, Bedford, MA) in DMEM medium were injected subcutaneously into the right flank of the mice. To determine suitable metformin and aspirin doses for our study, we carefully reviewed the available data. Based on our recent study [15] and other prior studies [10, 11, 59] we administrated metformin 200 mg/kg, and aspirin 60 mg/kg three times a week (Monday, Wednesday and Friday) by intraperitoneal (i.p.) injection. After 4–6 weeks, mice with PANC-1 tumors (0.5–1.0 cm wide and 0.5–1.0 cm long) were randomized and injected by i.p. with vehicle (5 *μ*L/g body weight), metformin (200 mg/kg), aspirin (60 mg/kg), or metformin (200 mg/kg) plus aspirin (60 mg/kg) three times a week for 28 days. The mice in the different experimental groups received the same amount of vehicle (5 *μ*L/g body weight) which consisted of propylene glycol, polysorbate 80, benzyl alcohol, ethanol and water (40:0.5:1:10:48.5) (14). Tumor size (length and width) and body weight were measured before each i.p. injection. All animal experiments were carried out under an Institutional Animal Care and Use Committee (IACUC)-approved protocol.

### Statistical analysis

The method by Laska *et al* [60], based on isobologram, was used to assess the synergistic effects of the combination of metformin with aspirin on cell viability. One-way ANOVA with post hoc Dunnett's test was used for assessing the treatment effects on colony information, cell migration, apoptosis, as well as the modulations of protein expression. The tumor volume was calculated by using the formula length × width × width/2. The analyses of percentage of initial tumor volume were based on a repeated measurement model [61]. The treatment effects were assessed by comparing the rates of change over time between treatment groups (i.e. comparing the slops and/or quadratic trends between treatment groups). Heterogeneous autoregressive correlation structure was used to account for the within-mice correlation. The ANOVA model with Tukey-Kramer adjustment [62] was used for the comparison of tumor volume and body weight among different treatment groups at the end of the treatment. A P value less than 0.05 was considered statistically significant.

## SUPPLEMENTARY MATERIAL FIGURES


