# Supplementary material for: Metformin combined with aspirin significantly inhibit pancreatic cancer cell growth in vitro and in vivo by suppressing anti-apoptotic proteins Mcl-1 and Bcl-2
Source: Oncotarget. 2015 May 12;6(25):21208–24. doi: 10.18632/oncotarget.4126 (PMC4673260; doi:10.18632/oncotarget.4126)
Supplement: Supplementary file 1 [file oncotarget-06-21208-s001.pdf]

Metformin combined with aspirin significantly inhibit pancreatic cancer cell growth *in vitro* and *in vivo* by suppressing anti-apoptotic proteins Mcl-1 and Bcl-2

Supplementary Material

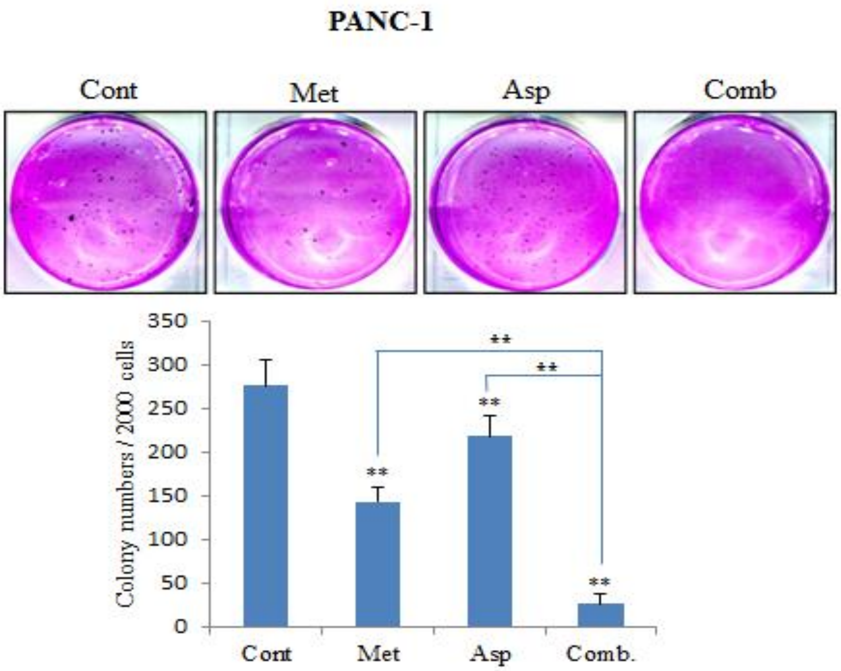

Supplementary Figure S1: Metformin and aspirin synergistically inhibit anchorage-independent colony formation. PANC-1 cells were treated with metformin (5 mM), aspirin (2 mM), or metfromin (5 mM) plus aspirin (5 mM) for 72 hours. The treated and untreated cells were trypsinized and mixed with soft agar. 2000 single viable cells were plated in 6-well plates and culture for additional 21 days. The colonies were stained with MTT and counted if they contain 50 cells or more. Data are means ± SEM, n = 3 means of triplicate measures. $^{*}p < 0.05$, $^{**}p < 0.01$, compared to the untreated control or individual drug. Cont, control; Met, metformin; Asp, aspirin; Comb, the combination of metformin and aspirin.

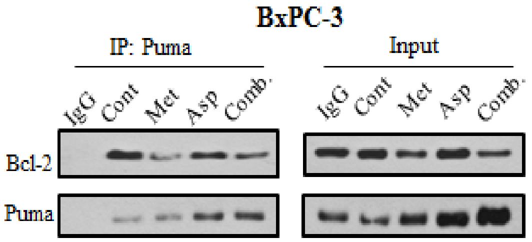

Supplementary Figure S2: Metformin and aspirin disrupt Bcl-2: Puma complexes in BxPC-3 cells. BxPc-3 cells were treated by indicated agents (metformin: 3 mM, aspirin: 1 mM) for 48 hours. Puma was immunoprecipitated from the whole cell lysates and analyzed for the presence of Bcl-2. Cont, control; Met, metformin; Asp, aspirin; Comb, the combination of metformin and aspirin.

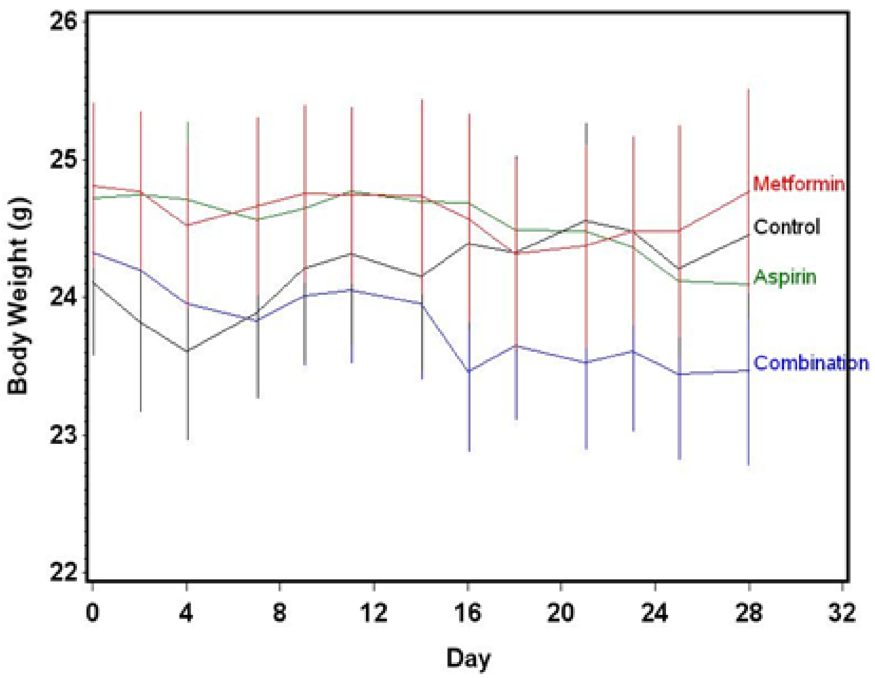

Supplementary Figure S3: No significant changes on the body weight of mice treated with metformin and aspirin. Mice (10 per group) with PANC-1 tumors (0.5–1.0 cm wide and 0.5–1.0 cm long) were randomized and injected by intraperitoneal (i.p.) with vehicle (5 µL/g body weight), metformin (200 mg/kg), aspirin (60 mg/kg), or metformin (200 mg/kg) plus aspirin (60 mg/kg) three times a week for 28 days. All mice were monitored daily for any discomfort, and body weight were measured before each i.p. injection. Values are means ± SE, n = 10.
